# Medical and Philosophical News

**Published:** 1781-04

**Authors:** 


To the account already given of the late M. Lieutaud, in our Journal for February (page 138) we are now enabled to add the following particulars of the life of that celebrated phyſician — Joſeph Lieutaud was born at Aix in 1703. He was the youngeſt of twelve children, and was naturally of a weakly conſtitution. He was educated under his uncle Gabriel Lieutaud, who acquired ſome reputation as a botaniſt. After taking a doctor's degree at Aix, he ſpent ſeveral years at Montpelier, and from thence returned to his native city, where he procured the appointments of Profeſſor of Phyſic, and Phyſician to an hoſpital, and ſoon came into extenſive practice. His houſe became the rendezvous of men of letters, who met there on ſtated days to converſe on literary ſubjects. The famous Marquis d'Argens uſed ufed often to affift at thofe meetings, and ever after profeffed a friendfhip for M. Lieutaud.

About the year 1750, he was called from Aix to Verfailles to act as phyfician to the Royal Infirmary, and this circumftance may be confidered as the foundation of his future good fortune. While he officiated in that capacity, he diffected a great number of bodies, and publifhed his obfervations on the heart and bladder. He was afterwards appointed phyfician to the princes of France, and in 1774 firft phyfician to the King, a poft not only of great honour, but of confiderable emolument, the profits of it being eftimated at upwards of £ 3000 fterling per annum. His uncle Gabriel had bequeathed him his library which was confiderable. M. Lieutaud, by the additions he made to it rendered it very valuable, and Monfieur, the King's eldeft brother, gave him a confiderable fum for it, and left him the free poffeffion of it during life. M. Lieutaud lived in a frugal and temperate manner, and died fuddenly.

Befides the works we formerly mentioned, he was author of the following; viz. *Elementa phyfiologiæ, juxta folertiora, noviffimaque phyficorum experimenta et accuratiores anatomicorum obfervationes,*

*servationes, concinnata.* Amstelodam. 8vo. 1749. *Precis de la matiere medicale*, 8vo. Paris, 1766. He was likewise the author of several anatomical papers, printed in the Memoirs of the Royal Academy of Sciences for 1752, 1753, and 1754.

